# Role of data mining in establishing strategic policies for the efficient management of healthcare system – a case study from Washington DC area using retrospective discharge data

**DOI:** 10.1186/1472-6963-12-S1-P12

**Published:** 2012-11-21

**Authors:** Harleen Kaur, Ritu Chauhan, Zafar Ahmed

**Affiliations:** 1 United Nations University International Institute of Global Health (UNU-IIGH), Kuala Lumpur, Malaysia; 2 Hamdard University, New Delhi, India; 3 International Case Mix and Clinical Coding Centre, UKM Medical Centre, Faculty of Medicine, University Kebangsaan Malaysia, Kuala Lumpur, Malaysia

## Background

Many physicians are perplexed with an acute increase in the rate of pregnancies and live birth, and a decrease rate of abortions among the women in the Washington DC area. Healthcare managers are analyzing the admission and discharge data to understand this trend. There are many factors such as marital status, age, income, health problems during pregnancy, insurance coverage and other treatment expenses, existence of psychological and emotional problems, patients experience while stay at hospital, and most importantly the increasing cost of abortion that can lead to this sudden increase in rate of live birth and subsequent decrease in the abortion rate in Washington DC area.

## Objective

The main objective of this study is to study the factors that lead to this increase in the rate of pregnancies and live birth, and decrease in the rate of abortion in the Washington DC area.

## Methodology

A qualitative approach is used to evaluate and determine the factors that have lead to this increased rate of pregnancies and live child births in Washington, DC area. The Data mining, clustering and statistical techniques were used to evaluate the Casemix datasets to understand the causes increased rate of pregnancies, live birth and reduce rate of abortions in Washington, DC area. Eight hospitals in Washington DC area were randomly selected and included in this study. The Casemix data set of the patient giving live birth at these eight hospitals were abstracted and studied from January to December 1992. The associated patterns leading to the increased rate of live birth and decrease rate of abortions are discovered for further analysis with K means clustering and other statistical techniques.

## Results

The cost of abortion is the main factor, among the positive factors, that has lead to an increase rate of live births among the cases studied. The unmarried women who cannot afford the cost of abortion were ranked among the highest to continue with the pregnancy whereas the rate of live birth was lowest among the married women’s.

## Conclusion

This type of information will provide the basis for the proper strategic planning and can help establish policies to provide assistance to the unmarried women’s for abortions or a different policy’s to remove the cost of burden for abortion. The study may help to develop policies that can lead to a decrease in the rate of live births due to increase in cost of abortion.

